# Emergency and urgent dental visits among Medicaid enrollees from 2013 to 2017

**DOI:** 10.1186/s12903-020-01345-7

**Published:** 2020-12-04

**Authors:** Rebekah Fiehn, Ilya Okunev, Mary Bayham, Steven Barefoot, Eric P. Tranby

**Affiliations:** 1 DentaQuest Partnership for Oral Health Advancement, 465 Medford Street, Boston, MA 02129 USA; 2 DentaQuest, 11100 West Liberty Drive, Milwaukee, WI 53224 USA

**Keywords:** Emergency dental, COVID-19, Teledental, Medicaid

## Abstract

**Background:**

Better understanding of the frequency of dental emergencies and the procedures performed during those emergency visits can help providers, insurers, and policymakers understand workforce and care provision needs.

**Methods:**

Procedures performed at an emergency dental encounter and in the encounter following that encounter are assessed. Emergency dental encounters are those with a CDT code of D0140, D0160, or D0170. Data was analyzed from the IBM Watson Medicaid Marketscan data from 2013 to 2017, a nationally representative dental and medical claims database from 13 deidentified states in the United States.

**Result:**

Consistently over time, about 10% of all dental encounters are due to a dental emergency. 28% of emergency dental encounters had no other procedure performed during those encounters. When other procedures were performed during the encounter, the majority were diagnostic in nature, primarily radiographs. Among patients who returned to the dentists following an emergency visit, 43% returned for more definitive dental treatment, most within 30 days.

**Conclusions:**

The majority of dental emergency encounters do not result in definitive treatment, rather patients often return to the dentist at a later date for that treatment. Where possible, dental providers could utilize teledental services to triage patients to appropriate care.

## Background

It is important for health care systems to have a general understanding of what constitutes a dental emergency to ensure that patients have access to essential care and establish best practice pathways for determining appropriate care location. Providers need to understand the critical elements of administering emergency dental care, the factors which may influence patients to seek out care, and plan appropriately for triaging and treating emergency cases. However, there is a gap in research around dental emergencies in dental settings. The research available in the United States has largely focused on pediatric dental emergencies including those originating from trauma [1, 2]. A study out of South Carolina found that just 9% of the after-hours pediatric dental emergencies analyzed needed referral to ED for treatment while the rest could be addressed in the dental setting. Additionally, the study found that there was significant variation in the treatment decisions partly due to unique provider characteristics (pediatric vs general) or practice settings [1]. Studies from outside of the US have mainly focused on the reasons for the emergency dental visit, with little emphasis on what happens following the emergency dental visit [3, 4].

In the United States, limited access and reductions in covered services by public health programs often lead to increases in emergency department (ED) services. A study of the emergency department visits at the University of Illinois Hospital found the reduction in dental benefits was followed by increases in ED visits (48%), surgical interventions (100%), and hospital admission days (128%) [5]. Most dental care in the ED is palliative and consists of infection management through antibiotics and pain management through analgesics. Most EDs are not equipped to provide definitive care for dental conditions such as dental pulpal or periapical lesions, cellulitis or abscess, injuries, and pain. ED interventions are directed toward treating symptoms of the underlying condition without resolving the primary issue which often leads to revisits and may lead to the over prescribing of opioids and antibiotics [6–12]. ED resources and care teams should be focused on the management of infectious and critically ill patients, so it is vital that dental emergencies are kept in dental settings where appropriate and definitive treatment can be established.

Better understanding of the frequency of dental emergencies and the procedures performed during emergency visits can help providers, insurers, and policymakers understand workforce and care provision needs both within and outside of the coronavirus disease 2019 (COVID-19) pandemic environment. To that end, a retrospective study of data of Medicaid claims from 2013 through 2017 is used to (1) identify trends in emergency dental visits, (2) describe what happens during emergency dental visits, and (3) identify common treatment pathways following emergency dental visits.

## Methods

This retrospective study used Medicaid claims data to study the prevalence and composition of Emergency Dental visits.

### Data source

This study used deidentified medical, dental, and pharmaceutical claims data from January 1, 2013 through December 31, 2017 from the IBM Watson MarketScan Multi-State Medicaid Database core data set [13]. This is a very large database with billions of records on millions of patients, has been used in hundreds of peer reviewed publications, and is generally accepted as a nationally representative dental and medical claims database of Medicaid data. That said, the data includes all Medicaid claims from 13 deidentified states and, therefore, are not drawn from a random sample. As such, findings are not guaranteed to generalize to the larger U.S. population [14].

### Sample selection

Emergency dental encounters are those with a Code on Dental Procedures and Nomenclature (CDT) code of D0140, D0160, or D0170, which are codes commonly used to identify limited exams (D0140), extensive (D0160) exams, or re-evaluations (D0170) done by a dentist in a dentist’s office or similar facility on an urgent or emergency basis. CDT codes are the standard set of procedural codes for oral health, commonly used for billing purposes, and widely available in claims data. Because the findings show consistency in trends over time, we often focus on a single year analysis, which is typically 2016. Procedures performed at an emergency dental encounter in 2016 are assessed, and are sometimes compared to procedures performed at non-emergency dental encounters in the same year. Follow-up encounters occurring between 1 and 365 days after the initial emergency dental encounter are also assessed. Follow-up encounters may occur at any time in 2016 or 2017.

We included all patients aged 0 to 64 with at least 1 dental claim in the year 2016 in the study. In line with Medicaid policy, patients aged 0 to 20 are defined as children. Patients aged 21 to 64 are defined as adults. These categories are based on eligibility for child versus adult Medicaid.

### Variables

We defined prescriptions as being associated with an emergency dental visit if the prescription started on the same day as an emergency dental visit, regardless of whether patients had other visits or not that day.

When comparing emergency dental visits to follow-up visits, the first emergency dental visit in the year for the patient is considered as the index emergency visit. The follow-up visit by the patient may be another emergency dental visit, a treatment visit or a routine office visit. A treatment visit in this case is defined by procedure codes D2XXX–D7999, which includes restorative procedures, oral surgeries, and endodontic procedures, while a routine office visit is considered to be any non-emergency, non-treatment visits to a dentist, incorporating routine examination, diagnostic services, and preventive care.

In some parts of the analysis, we used the integrated nature of the claims database and the detailed information on the timing of visits to examine the correlation between emergency dental encounters in the dental office and visits to the hospital emergency room (ER) for dental conditions. Dental conditions are defined using the ASTDD classification of ICD-10 diagnostic codes for non-traumatic dental conditions We examined ER visits that occurred within 15 days of both emergency and non-emergency dental visits (visits that occurred at a dentist’s office). We are also examined the number of emergency dental and ER visits that occurred within 1 year of the emergency dental visit.

## Results

The analysis is derived from data of over 12 million Medicaid enrolled members in 2016 (with enrollment totals ranging from 11 million in 2013 to 13.5 million in 2017), of whom 3.9 million visited a dentist and 649,442 had an emergency dental visit (Table 1). The demographic characteristics of the data used in this analysis are similar to the results of a nationally representative sample of Medicaid enrollees, with the exception of racial characteristics, where our sample had more Blacks and fewer Hispanics than the nation as a whole.

**Table 1:** Demographic characteristics of study population (in 2016)

	Variable	Medicaid enrolled population (IBM Watson Data)	Nationally representative sample of Medicaid enrolled population	Population with at least one dental visit (IBM Watson)	Population with one or more emergency dental visits (IBM Watson)
	Count	% within categories	% within categories	% within categories	% within categories
Sex					
Male	5,721,884	43	44	46	39
Female	7,571,087	57	56	54	61
Race					
White	6,436,589	47	40	47	51
Black	4,245,692	32	19	32	30
Hispanic	704,674	9	29	9	6
Other	1,906,016	12	12	12	13
Age					
0–10	4,054,204	33	30	46	29
11–20	2,955,882	24	25	32	24
21–34	2,220,470	18	18	10	21
35–51	1,684,552	14	15	8	16
51–64	1,329,492	11	12	5	11
Total	12,244,600	100	100	32 had dental visit	5 had emergency dental visit

National estimates of the Medicaid enrolled population are derived from the Medical Expenditure Panel Survey (2016)

**Fig. 1:**
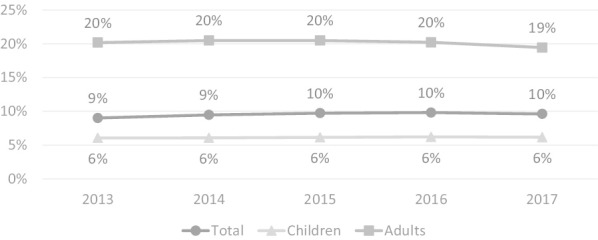
Proportion of all dental encounters among Medicaid enrollee’s that are emergencies from 2013 to 2017, including in the population overall (circles), among children ages 0–20 (triangles), and adults 21 or older (squares)

**Fig. 2:**
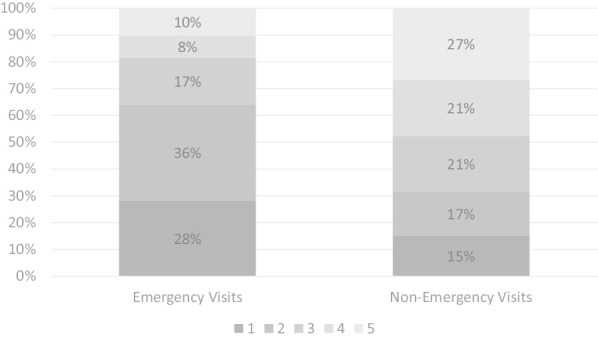
Number of dental procedures performed during a dental visit, comparing emergency visits on the left and non-emergency visits on the right

Out of all dental encounters in 2016, 10% were emergency dental encounters. When assessed by age group, emergency encounters accounted for 6% of total encounters for children and 20% for adult encounters (Fig. 1). These numbers were nearly identical from 2013 to 2017 (Fig. 1), demonstrating stability in these estimates over time. 28% of emergency dental encounters had no other procedure performed during those encounters, other than the emergency visit code, while another 36% had only one additional dental procedure performed during the visit (Fig. 2). Most additional procedures performed during an emergency dental visit were related to radiographs or intraoral images, comprising 61% of all procedures on children and 68% of all services performed on adults. The next most common procedure category was oral surgeries, predominantly extractions, comprising 12% of all services performed on children and 17% of all services performed on adults (See Table 2, See Additional file 1: Table S1 for the most common CDT codes).

**Table 2:** Procedure code groupings

Grouping	Children		Adults	
	Emergency visit (%)	Visit after emergency visit (%)	Emergency visit (%)	Visit after emergency visit (%)
Imaging	61	23	68	28
Oral surgery	12	9	17	23
Anesthesia	7	8	2	4
Minor restorations	7	9	6	13
Preventive	5	28	1	8
Major restorations/endodontics	4	6	2	4
Adjunctive general	2	3	2	2
Diagnostic	1	15	1	13
Periodontics	0.3	0.1	1	2
Prosthodontics/orthodontics	0.1	0.6	1	3

**Fig. 3:**
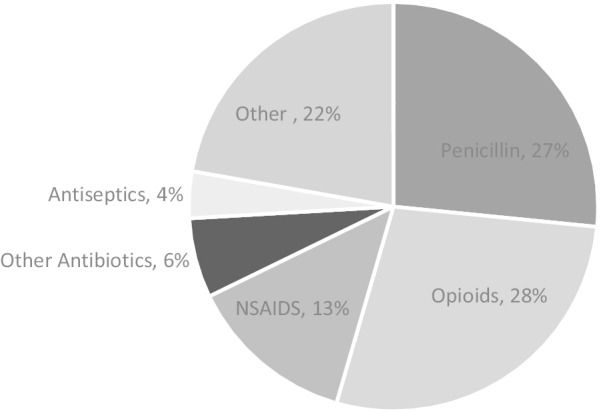
Type of medication prescribed on the same day as an emergency dental visit, defined by therapeutic class of drug

36% of adult dental emergency visits had a prescription associated with them and 19% of visits by adults were associated with an opioid prescription. Among children, 15% of visits had an associated prescription and 4% had an associated opioid prescription. The most common prescription category prescribed for all emergency dental visits was Opioids at 28% of all prescriptions. The next most common prescription category was Penicillin with 27% followed by NSAIDS with 13% (Fig. 3).

Of all patients with an emergency dental visit in 2016, 78% returned to the dentists within 365 days of their first emergency dental visit. Among those patients that return, we see significantly fewer imaging procedures as a percent of total and more preventive and treatment (Table 2, Additional file 1: Table S2). Among children, imaging as a percent of total, dropped from 60 to 23% between their first emergency dental visit and their next follow-up. For adults, imaging dropped from 68 to 28%. Preventive services, in children, went up from 5.4 to 28.2%. Restorations (both Minor and Major), as a percent of total, went up from 10.9 to 14.5% in children and from 7.8 to 17.4% in adults.

Categorizing return visits, 19% were for another emergency dental visit, 43% were for dental treatment, such as a restorative or surgical procedure, and 39% were for routine dental care, such as preventive care (Table 3). Patients are most likely to return for a follow-up within 15 days of an emergency dental visit if the follow-up visit is a treatment visit, as 44% of treatment visits occurred in this time frame. Meanwhile, 32% of return visits that were also emergency visits occurred within 15 days. Only 16% of return routine visits occurred within 15 days, and nearly half occurred 90 or more days after the dental emergency.

**Table 3:** Days to return visit within 365 days after emergency visit, by visit type

	Another emergency visit (19% of return visits) (%)	Treatment visit (43% of return visits) (%)	Routine visit (39% of return visits) (%)
1–15	32	44	16
16–30	15	20	11
31–60	15	17	14
61–90	9	7	11
91–180	15	8	27
181–365	14	4	20
Total	100	100	100

Emergency dental visits at a dentist’s office can lead to emergency room (ER) visits for dental conditions, as patients are more likely to visit the ER for a dental condition within 15 days of a dental visit if the dental visit was classified as an emergency dental visit. 1.7% of adult emergency dental visits resulted in an ER visit for a dental condition within 15 days (0.6% in children), compared to 0.6% for non-Emergency adult dental visits (0.1% in children). The emergency dental to ER pathway can lead to a vicious cycle of visits, likely due to unresolved issues, as 3.4% of emergency dental visits among adults (1% among children) occur within 15 days of an ER visit for a dental condition. Additionally, half of patients who have both an emergency dental visit and an ER visit for a dental condition have 2 or more emergency dental visits within 365 days, while slightly more than half have 2 or more ER visits for dental conditions within 365 days.

## Discussion

Dental emergency visits tend to be evaluative in nature or are associated with prescriptions for pain or infection management, rather than providing definitive treatment, as more than 1 in 4 visits had no other procedure associated with them and, when there are other procedures, they tend to be for imaging and other non-definitive treatments. Dental emergency visits, however, are often a pathway to more definitive dental treatment, with 65% of those who return for dental treatment coming back within 30 days. This finding is in line with contemporary research during the COVID-19 pandemic in the UK, which found that 65% of urgent dental visits lead to definitive dental treatment and that emergency dental visits can effectively triage patients into needed clinical care [15].

Emergency dental visits, alone, are not enough for keeping people out of the ER for dental conditions, as about 2% of adults who have an emergency dental visit end up going to a hospital emergency room within 15 days. Rather, a full range of preventive and dental treatments are needed, as emergency dental visits are more often associated with ER visits than non-emergency visits and there can be a vicious cycle of emergency dental and ER visits for dental conditions. Among those enrolled in Medicaid, there is a great deal of stability over time in the rate of emergency dental visits with about 10% of dental encounters being on an emergency basis.

Efforts to stem the spread of SARS-CoV-2 and COVID-19 led to restricting the provision of dental care to dental emergencies. Recommendations to limit routine dental care by the WHO were adopted by many countries [16]. While the response by regulatory and governing bodies has varied, much of the initial focus was on reducing viral spread, ensuring patient and provider safety and allowing time for updates to infection control policies and practice through limiting dental services [17–22].

In the United States of America, the response to COVID-19 from the Centers for Disease Control and Prevention (CDC), a national public health institute in the United States, along with the nation’s largest dental association, the American Dental Association (ADA) included initial guidance which encouraged limiting dental care to urgent or emergent treatment [23, 24]. ADA interim guidance on returning to provide non-emergent care urged that treatment should be decided on patient or community risk of COVID-19, clinical risks associated with aerosol generating procedures, and the availability of personal protective equipment[25].

Dental professionals are at considerably high risk of exposure to pathogenic microorganisms that infect the oral cavity and respiratory tract due to the nature of the dental care setting and procedures, which involve a face-to-face proximity between patient and provider, handling of high-speed handpieces, and exposure to saliva, blood, and other body fluids [26–29]. The COVID-19 crisis and the resulting dental service restrictions presented providers, payers, and patients with an unprecedented challenge, and determining the full impact on overall oral health and long-term changes in demand for services will be difficult to predict. These reductions in dental services, even for a short period of time, will have significant impact on the oral health of Americans. Recent analysis has shown that 92% of families in poverty or low incomes have unmet dental needs [30]. These families rely on public insurance programs and access to low-cost or free dental services to address their needs. Given the great burden of dental disease in these populations, limitations on scope of service and dental office closures across the country have had a disproportionate impact on individuals experiencing poverty, the uninsured, and individuals who participate in United States government-sponsored programs such as Medicaid, which helps cover health care costs for low-income Americans, generally under the age of 65 [31].

There are several limitations to this study. While the dataset used is a large database with a population that is consistent with the Medicaid population as whole, it is not randomly generated sample of all Medicaid claims in the United States and so should not be assumed to be definitively generalizable. Additionally, while we focused on the Medicaid population to both the lack of information on this population and their unique vulnerability to untreated dental conditions, the Medicaid population is not like the population as a whole in the United States. Due to the consistency in trends over time, this study focuses on a single year of data for the bulk of the analysis and so cannot definitely state that the findings hold across all years of data nor that the patterns will hold across time. Regardless of the limitations, we believe that the findings reported here, including the stability of emergency dental visits over time, the nature of those visits as predominately evaluative in nature, and the outcomes of those visits for patients as often resulting in definitive treatment, but that they can lead to a cycle of emergency dental and hospital utilization for a small subset of patient provide useful information that can be built on by future work and used to make decisions in the current environment.

## Conclusion

Given what is known about the infection pathways of COVID-19 and with a general understanding of emergency dental visits in the dental setting, providers can effectively plan and determine how to provide essential care to patients while protecting themselves, staff, and the individuals who are seen for treatment. The provision of care under the threat of COVID-19 or other air-borne infections will require the adoption of new techniques for infection control in dental settings. Additional emphasis on the reduction of aerosols may result in increased usage of non-aerosol generating treatments for carious lesions, such as silver diamine fluoride, temporary restorations with glass ionomers, or atraumatic restorative treatments [32–34]. Providers should be prepared for evolving guidance and continued adjustments to the way they practice, as evidence-based research is established for COVID-19 and dental treatment.

Significant efforts have been made in providing and examining infection control and clinical management of dental emergencies. While many practices have reopened with increased infection control procedures, the pandemic is not well-controlled in many regions of the United States and there may be ongoing delays in people receiving dental care or even additional shutdowns of dental care [35]. Remote triage tools, such as teledentistry can assist in keeping providers and patients engaged and communicating effectively throughout the changes in operations and may be an essential tool in the transition to a new era in dentistry, assuming that regulatory and financial barriers to implementation are addressed in an expedient manner [36]. Through teledentistry and in collaboration with patients, providers can engage in more patient outreach, reinforce healthy behaviors, provide education, and explore minimally invasive treatment options without being in physical proximity. Additionally, providers who connect with patients via telehealth can triage and direct care to appropriate settings, maximizing the productive use of patient time in the operatory while reducing exposure risk. As a result of the COVID-19 pandemic, providers have the opportunity to refocus care delivery to be more agile in responding to future closures, reducing the demand for emergency services through pathway redesign, and moving from a “drill first” focus to prevention-focused built around the most minimally invasive procedures possible.

## Supplementary information

**Additional file 1.** Top 20 Procedure Codes During Emergency Visits and Visit after an Emergency Visit.

## Data Availability

The data that support the findings of this study are available from International Business Machines (IBM), but restrictions apply to the availability of these data, which were used under license for the current study, and so are not publicly available. No administrative permissions are required to use this data, however a licensing fee for use of the data is generally required. Data may be available from the authors upon reasonable request and with permission of IBM.

## Abbreviations

ADA: American Dental Association
ASTDD: Association of State and Territorial Dental Directors
CDC: Centers for Disease Control and Prevention
CDT: Code on Dental Procedures and Nomenclature
CMS: Centers for Medicare and Medicaid Services
COVID-19: Coronavirus disease 2019
ER: Emergency room
FQHC: Federally Qualified Health Centers
HRSA: Health Resources and Services Administration
ICD: International Statistical Classification of Diseases and Related Health Problems
MUA: Medically underserved areas
