# Supplementary material for: Emergency and urgent dental visits among Medicaid enrollees from 2013 to 2017
Source: BMC Oral Health. 2020 Dec 4;20:355. doi: 10.1186/s12903-020-01345-7 (PMC7716286; doi:10.1186/s12903-020-01345-7)
Supplement: Supplementary file 1 — Additional file 1. Top 20 Procedure Codes During Emergency Visits and Visit after an Emergency Visit. [file 12903_2020_1345_MOESM1_ESM.docx]

**Supplemental Tables**

| **Supplemental Table S1: Top 20 Procedure Codes During Emergency Visits (Each procedure code is counted once per visit)** | | | |
| --- | --- | --- | --- |
| **#** | **Procedure Code** | **Visits** | **% of Total** |
| 1 | D0220 | 385,452 | 36.9% |
| 2 | D0330 | 117,691 | 11.3% |
| 3 | D0230 | 92,027 | 8.8% |
| 4 | D7140 | 84,608 | 8.1% |
| 5 | D7210 | 32,481 | 3.1% |
| 6 | D9230 | 30,045 | 2.9% |
| 7 | D0272 | 24,709 | 2.4% |
| 8 | D0274 | 17,979 | 1.7% |
| 9 | D0270 | 13,547 | 1.3% |
| 10 | D2392 | 13,357 | 1.3% |
| 11 | D0240 | 12,188 | 1.2% |
| 12 | D9110 | 10,604 | 1.0% |
| 13 | D2391 | 10,009 | 1.0% |
| 14 | D2335 | 9,993 | 1.0% |
| 15 | D9223 | 9,771 | 0.9% |
| 16 | D7240 | 7,671 | 0.7% |
| 17 | D2393 | 6,962 | 0.7% |
| 18 | D0210 | 6,811 | 0.7% |
| 19 | D1206 | 6,245 | 0.6% |
| 20 | D7111 | 6,206 | 0.6% |

| **Supplemental Table S2: Top 20 Procedure Codes During Visit after Emergency Visit (Each procedure code is counted once per visit)** | | | |
| --- | --- | --- | --- |
| **#** | **Procedure Code** | **Visits** | **% of Total** |
| 1 | D0120 | 97,737 | 8.7% |
| 2 | D0220 | 85,596 | 7.6% |
| 3 | D1120 | 76,438 | 6.8% |
| 4 | D7140 | 71,699 | 6.4% |
| 5 | D0150 | 54,867 | 4.9% |
| 6 | D1208 | 53,967 | 4.8% |
| 7 | D0272 | 53,380 | 4.8% |
| 8 | D1110 | 49,788 | 4.4% |
| 9 | D0230 | 44,897 | 4.0% |
| 10 | D1206 | 39,850 | 3.5% |
| 11 | D0274 | 37,399 | 3.3% |
| 12 | D0330 | 37,295 | 3.3% |
| 13 | D9230 | 33,687 | 3.0% |
| 14 | D7210 | 32,900 | 2.9% |
| 15 | D2392 | 26,907 | 2.4% |
| 16 | D2391 | 21,166 | 1.9% |
| 17 | D2930 | 17,782 | 1.6% |
| 18 | D9223 | 17,150 | 1.5% |
| 19 | D7240 | 15,291 | 1.4% |
| 20 | D1351 | 12,675 | 1.1% |
